# Emergency Management of Spontaneous Bacterial Peritonitis – A Clinical Review

**DOI:** 10.7759/cureus.2253

**Published:** 2018-03-01

**Authors:** Tracy MacIntosh

**Affiliations:** 1 Emergency, Osceola Regional Medical Center

**Keywords:** spontaneous bacterial peritonitis, cirrhosis, infection, sepsis, fever

## Abstract

Spontaneous bacterial peritonitis (SBP) has a high mortality rate; early antimicrobial therapy is essential for improving patient outcomes. Given that cirrhotic patients are often coagulopathic, the perceived risk of bleeding may prevent providers from performing a paracentesis and ruling out this potentially deadly disease.

We examine the pathophysiology and risk factors for SBP, and current guidelines for its diagnosis and treatment. We then review the time-sensitive nature of performing a paracentesis, and the current controversies and contraindications for performing this procedure in patients at risk for SBP.

Cirrhotic patients with ascites and clinical suspicion for SBP—abdominal pain or tenderness, fever or altered mental status—should have a diagnostic paracentesis. Although most patients with cirrhosis and liver dysfunction will have prolonged prothrombin time, paracentesis is not contraindicated. Limited data support platelet administration prior to paracentesis if <40,000-50,000/μL. Timely antimicrobial therapy includes a third-generation cephalosporin for community-acquired infection; nosocomial infections should be treated empirically with a carbapenem or with piperacillin-tazobactam, or based on local susceptibility testing. Patients with gastrointestinal (GI) hemorrhage should receive ceftriaxone prophylactically for GI hemorrhage.

SBP has a high mortality rate. Early diagnosis and antimicrobial therapy are essential for improving patient outcomes. Cirrhotic patients with ascites with clinical suspicion for SBP, abdominal pain or tenderness, altered mental status or fever should have a diagnostic paracentesis performed prior to admission unless platelets <40,000-50,000/μL.

## Introduction and background

Between 2006 and 2011, there were a total of 3,127,986 cirrhosis-associated emergency department (ED) visits in the United States. During this period, spontaneous bacterial peritonitis (SBP) represented up to 4% of these visits [1]. The United States has been experiencing an increasing incidence of hepatitis C, with approximately 30,000 cases in 2013, of which 75-85% of cases may become chronic and up to 20% may develop cirrhosis [2].

SBP is one of the most common infections in hospitalized patients with cirrhosis. A prospective study found that 47% of hospitalized patients with cirrhosis had bacterial infections, of which SBP was the most common, representing 31% of these infections [3]. Early recognition and treatment are essential for reducing the mortality associated with this condition.

One of the first challenges of diagnosis and treating SBP early in the ED is to be able to safely perform a paracentesis on a population with a high prevalence of coagulopathy and thrombocytopenia. Second, the appropriate and timely administration of antibiotics is essential for improving outcomes. This systematic review of the literature provides a foundation for understanding the etiology and clinical presentations of SBP. Best practice recommendations are given on when and how to perform a paracentesis, and on antibiotic treatment.

## Review

Etiology and pathophysiology

Cirrhosis is the end-stage of chronic liver disease, and is most commonly caused by hepatitis C infection and alcohol abuse, with an increasing burden from non-alcoholic liver disease [4]. It is classified as compensated if asymptomatic with normal or low portal pressures, and becomes decompensated once portal pressures increase, progressing to liver dysfunction and the development of ascites (most common sign to appear), varices, encephalopathy and/or jaundice [5]. The incidence of compensated progressing to decompensated cirrhosis varies depending on the underlying etiology: overall, the progression occurs in 42% of patients at 10 years and 62% at 20 years. In one study, 60% of patients with alcoholic cirrhosis progressed to decompensated cirrhosis, compared to 16% in patients with hepatitis C, and 32% of patients with both alcoholic and hepatitis C cirrhosis. New treatments for hepatitis C will most likely decrease the incidence further [6]. Patients with decompensated cirrhosis with ascites have a 20% one-year mortality, increasing to 57% if they have both ascites and variceal bleeding [5].

Liver dysfunction causes abnormalities in host defense. Patients with cirrhosis are more vulnerable to bacterial translocation due to intestinal bacterial overgrowth, increased intestinal permeability, and altered immune defenses, thereby increasing susceptibility to infection, particularly SBP [7]. Patients with cirrhosis and portal hypertension have increased intestinal permeability, resulting in bacterial and endotoxin translocation to mesenteric lymph nodes and other external sites [8].

The most common organisms in SBP are gram-negative bacteria, including E. coli. In a well-done meta-analysis including 7,062 patients with SBP, Arvaniti, et al. found that SBP mortality was 32% at one month, 26% at three months, and 60% at 12 months [9]. SBP complicates 25-65% of patients with cirrhosis who have a gastrointestinal (GI) hemorrhage, particularly with advanced disease or severe bleeding [10]. Patients with bacterial infection (including SBP) are at increased risk of failure to control bleeding [11], rebleeding, and mortality [12].

Differential diagnosis

The differential diagnosis in a patient suspected of having SBP lies in maintaining a broad suspicion for other conditions that cause abdominal pain and fever in a patient with a history of, or clinical suspicion for, ascites or cirrhosis.

Ascites

The vast majority (84%) of ascites is due to cirrhosis; however, in the absence of cirrhosis, most cases of ascites will be due to peritoneal carcinomatosis, cardiac ascites, peritoneal infection, or pancreatic illness. Less common causes include tuberculous peritonitis, hepatic metastasis, other infectious, pancreatitis, and nephrotic syndrome [13].

Fever

Up to 50% of patients with cirrhosis will present with a bacterial infection necessitating hospital admission, with a mortality rate of 25% [14]. In a 2003 retrospective study of hospitalized patients with cirrhosis, SBP and urinary tract infections were the most common infections encountered, followed by pneumonia, skin and soft tissue infections, and bacteremia [15].

Emergency department evaluation

Clinical History

There are critical pieces in the history that can help guide the physician towards the diagnosis of SBP, and should be obtained from the patient, family or paramedics depending on the patient’s ability to provide a history. Chinnock, et al. conducted a prospective, observational study of 144 ED patients receiving a paracentesis to determine the predictive ability of clinical characteristics and physician assessment for SBP. A history of fever in the last 24 hours was 81% specific for SBP, but was not useful in differentiating SBP from other sources of infections. The absence of abdominal pain was 94% sensitive for ruling out SBP. Physician’s clinical judgment demonstrated a limited sensitivity of 77% and specificity of 34% for detecting SBP [16].

While providing important insights into the clinical diagnosis of SBP, this study did have patient selection bias as they enrolled patients who the physician believed warranted a paracentesis, and therefore represented patients with a higher a priori suspicion for SBP. Given that this study demonstrated only modest sensitivity for physician clinical judgment for diagnosing SBP, it is unknown what impact this may have had on patient selection and results. Overall, this study still provides clinicians with important data to use at the bedside to determine the likelihood of SBP in cirrhotic patients.

Physical Examination

Beginning with an accurate set of vital signs, the presence of fever is highly specific for SBP among patients with ascites who receive a paracentesis. Tachycardia and tachypnea also alert the physician to the possibility of infection and sepsis. If the patient presents with altered mental status and evidence of cirrhosis and ascites, this raises suspicion for sepsis and infection, and is also highly specific for SBP in this population. Examine the patient for stigmata of chronic liver disease, including scleral icterus, jaundice, spider angiomas, gynecomastia, palmar erythema, ascites, encephalopathy, and asterixis.

Abdominal pain and tenderness are highly sensitive for SBP (94%). Altered mental status is also highly sensitive for SBP, and SBP should, therefore, be considered in the evaluation of altered mental status, along with hepatic encephalopathy. Fever on presentation, or in the last 24 hours, is highly specific for SBP (Table 1) [16].

**Table 1:** Sensitivity and specificity of selected clinical characteristics for SBP. SBP: Spontaneous bacterial peritonitis. Adapted from [16].

Clinical characteristic	Sensitivity	Specificity
Fever in last 24 hrs	35.3 (14.2-61.7)	81.1 (73.2-87.5)
Fever on exam (>38C)	17.7 (3.8-43.4)	90.1 (83.3-94.8)
Tachycardia (HR > 100)	56.3 (29.9-80.3)	47.9 (38.8-57.2)
Altered mental status	11.8 (1.5-36.4)	95.3 (90-98.3)
Any abdominal pain/tenderness	94.1 (82.9-100)	15.1 (8.8-21.3)

Diagnostic studies

Laboratory Studies

Patients with advanced liver disease and cirrhosis will generally have many laboratory abnormalities, a few of which have demonstrated predictive value for SBP, but are not diagnostic for SBP. Shi, et al. used a classification and regression tree model to identify a prediction model from general laboratory results to risk stratify patients predicting low, intermediate, and high risk of SBP with 88% accuracy. The higher risk groups had elevated serum Cr > 79.5 micromol/L, total bilirubin > 63.5 micromol/L, PT > 22.95, and white blood cells (WBCs) > 6,850/μL, though no variable demonstrated independent predictive value [17]. While still lacking external validation, this tool has the potential to allow physicians some insight for risk stratification. The utility of additional laboratory tests is discussed below:

Complete blood count: Patients may be anemic due to GI bleed, malnutrition, hypersplenism, or continued alcohol abuse. Portal hypertension and hypersplenism also lead to thrombocytopenia. Thrombocytopenia (<100,000/μL) has been shown to be predictive of SBP [18].

Bilirubin: Total bilirubin can be normal or elevated in advanced cirrhosis. Prothrombin times are usually prolonged. 

Complete metabolic panel: Sodium levels may be decreased in patients with ascites due to excess free water intake. Aspartate aminotransferase (AST) and alanine aminotransferase (ALT) levels are generally elevated in cirrhotics, particularly those with continued alcohol abuse [19]. Serum creatinine (Cr) levels may also be elevated in patients with cirrhosis as acute kidney injury is common in this population, and may be due to many causes including hepatorenal syndrome, shock, nephrotoxic medications, or intrinsic kidney disease [20]. While these laboratory abnormalities are common in patients with cirrhosis and ascites, they are neither sensitive nor specific for the diagnosis of SBP.

C-reactive protein (CRP): An elevated CRP > 60 mg/L demonstrated very high specificity for SBP (96.5%) in one study [18], but likely represents overall inflammatory response to infection, rather than specificity for SBP over other sources of infection.

Lactic acid: There is no established role for serum lactic acid in the diagnosis or prognostication of SBP beyond its demonstrated utility in severe sepsis and septic shock [21].

Urinalysis: Include a urinalysis in any patient as guided by the history and physical exam, with particular focus on patients with fever.

Paracentesis

The most important diagnostic study of any patient being evaluated for SBP or being admitted to the hospital with ascites and cirrhosis is a paracentesis. In a US national inpatient study of over 31,000 patients admitted with cirrhosis and ascites where about one-half underwent a paracentesis, the mortality rate for patients who underwent a paracentesis was significantly lower than for those who did not have one (6.3% vs 8.9%). This study highlights the adverse impact of a missed diagnosis of SBP, and how timely intervention can have a significant impact on mortality [22]. Furthermore, the timing of paracentesis is also key to reducing mortality rates. Kim, et al. performed a retrospective analysis of 239 patients with SBP and demonstrated that early paracentesis, performed within 12 hours, was associated with lower in-hospital mortality compared to delayed paracentesis, 12-72 hours (13% vs 27%), as well as shorter intensive care unit and hospital stays. The authors found that each hour of delay was associated with a 3.3% increase in in-hospital mortality, and early paracentesis (within 12-24 hours versus 48 hours or more) [23].

Peritoneal infection causes an inflammatory reaction and increasing number of neutrophils in ascetic fluid. Today’s guidelines were developed in the 1980s. Ascitic fluid polymorphonuclear (PMN) count >500/mL is the most specific for diagnosing SBP [10,24] though guidelines use a cut-off of >250/mL for greater sensitivity, and SBP is diagnosed in the absence of another intra-abdominal source of infection or malignancy.

According to the American Association for the Study of Liver Diseases (AASLD), “If ascitic fluid infection is suspected, ascetic fluid should be cultured at the bedside in aerobic and anaerobic blood culture bottles prior to initiation of antibiotics (Class 1, Level B),” noting that bacterial growth occurs 80% of the time when collected in culture bottles, compared to only 50% when collected in other containers [25]. Because ascitic fluid can be culture negative even when appropriately collected, it is not necessary for the diagnosis. Conversely, a positive culture should always be treated in a symptomatic patient, even if PMN < 250/mL. Asymptomatic patients with positive cultures should have a repeat cell count and culture, and be treated if PMN > 250/mL, otherwise follow-up is appropriate [10].

The ideal anatomical location for performing a paracentesis has not been established. The optimal location is one that avoids the inferior epigastric artery and has deep pocket of fluid, affording the greatest safety from bowel perforation. Sakai, et al. compared abdominal wall thickness and fluid depth among 52 patients and found that the left lower quadrant was superior to the infraumbilical midline position [26].

Ultrasound-guided paracentesis has been demonstrated to decrease the risk of bleeding (see Table 2) [27], and allows the clinician to identify adequate fluid, other pathologies, and change locations [28]. The presence of septations or debris in ascites fluid can be suggestive of infected ascites [29]. Ultrasound-guided paracentesis has demonstrated an excellent safety profile. In a study of 410 abdominal paracenteses, only two procedures (on the same patient) developed a minor complication, and there were no procedure-related bleeding and complications even among patients with marked thrombocytopenia (<19) or prolonged international normalized ration (INR) (>3.0) [30]. Similar results were demonstrated on patients with INR > 1.6 and/or platelets <50,000/μL who all had ultrasound-guided thoracentesis performed; patients either had their coagulopathy corrected with fresh frozen plasma (FFP) or platelets or not at all. A total of 1,009 procedures were analyzed with a total of four hemorrhagic complications (0.4%), and there was no difference in complication rate between the two groups [31].

The vast majority of paracenteses occur without any complications, but in the 10% of cases with complications, these are generally minor and self-limited, including self-limited bleeding and self-limited ascites fluid leak [32]. The introduction of iatrogenic infection is extremely rare, 0.2%, and bowel perforation remains a rare complication occurring in 0.8-2% of procedures [32,33], though specific studies are needed to compare rates with and without ultrasound guidance (Table 2).

**Table 2:** Risk of bleeding complications in patients undergoing paracentesis. Adapted from [27].

Exposure group	No. of events	No. at risk	%	95% confidence interval
All patients	565	69,859	0.81	0.77-0.84
Ultrasound guidance	87	31,649	0.27	0.26-0.29
No ultrasound guidance	478	38,210	1.25	1.21-1.29

Treatment

Early initiation of antimicrobial therapy is associated with improved outcomes. In a retrospective review of 126 cirrhotic patients with septic shock due to SBP, Karvellas, et al. found that every one hour delay in administering antimicrobial therapy in patients was associated with a 1.86 times increase in-hospital mortality [34]. Current guidelines recommend that community-acquired infections without recent β-lactam antibiotic exposure should be treated with a third-generation cephalosporin [14]; however, a recent German study found that third-generation cephalosporins would have only covered 70% of community-acquired SBP and 56% of nosocomial cases of SBP, and patients with resistant organisms had lower survival rates [35].

Given increasing prevalence of multi-drug resistant bacteria, broad-spectrum antimicrobial agents, such as carbapenems and/or glycopeptides or piperacillin-tazobactam should be considered [25]. Furthermore, there is increasing prevalence of methicillin-resistant Staphylococcus aureus (MRSA) infections among nosocomial SBP [35], representing 19-48% of culture-positive results [36]; therefore, if using third-generation cephalosporins based on current guidelines, careful patient re-evaluation for treatment failure for community-acquired infection is essential, and nosocomial SBP should receive empiric antibiotics based on local susceptibility testing of bacteria (Table 3) [14].

**Table 3:** Current antibiotic recommendations for SBP. SBP: Spontaneous bacterial peritonitis.

Medication	Specific indications
Third generation cephalosporin	Community-acquired infection
Carbepenems +/- glycopeptides Piperacillin-tazobactam	Nosocomial infections, patients hospitalized within past 90 days, long-term norfloxacin prophylaxis, history of multi-resistant bacterial infection, recent beta lactam use

Albumin plus antibiotics has been demonstrated to reduce the incidence of renal impairment and reduce in-hospital mortality from 29% to 10% [37], compared to antibiotics alone. A recent Cochrane review similarly supports the use of albumin for SBP and demonstrated protective effects on renal function and mortality [38]. Specific future studies stratifying results by pre-existing kidney function may reveal whether albumin administration should be tailored to pre-existing renal function or is protective to all cases of SBP, but based on the existing data, the administration of albumin for renal protection of patients with SBP is currently recommended by the current AASLD guidelines as a level B recommendation. Patients with SBP and a serum Cr > 1 mg/dL, BUN > 30 mg/dL or total bilirubin > 4 mg/dL should be given 1.5 mg/kg albumin within 6 hours of diagnosis, and an additional 1.0 mg/kg on day 3 (Class IIa, Level B) [25].

Prophylaxis

Infections are relatively common among cirrhotic patients with GI hemorrhage, and complicate 22% of upper GI bleeds. The most common infections are septicemia and SBP, and infected patients have higher mortality rates from GI bleeds compared to patients without infections [39]. Cirrhotic patients with GI bleed who received prophylactic antibiotics had significantly fewer cases of infections [40]. Therefore, prophylactic antibiotics, ceftriaxone IV 1 g/day for seven days, are indicated for all cirrhotic patients with active GI bleed [41].

Controversies and contraindications for paracentesis

Thrombocytopenia and Coagulopathy

Given that the majority of patients with cirrhosis and liver dysfunction will have some degree of thrombocytopenia and coagulopathy, the existing data indicate that paracentesis is not contraindicated in these patients, with the possible exception of severe thrombocytopenia. There is no data to support the use of FFP prior to paracentesis [42], but consider giving platelets prior to paracentesis if the platelet count is <40,000-50,000/μL [43, 44]. In a retrospective study of 304 paracenteses performed in patients with platelets <50,000μL, there was a 1% rate of major bleeding requiring blood transfusion, and these three patients had platelets between 41,000 and 46,000/μL [45]. Similarly, in a study of 171 patients undergoing a total of 515 paracenteses, the rate of major complications from paracentesis was 1.6%, the most common of which were bleeding and infection. Minor, self-limited, complications occurred in 8.9% of cases, and included leakage of ascitic fluid at puncture site and local bleeding. Platelet count > 50,000 and INR were not associated with complications in the multivariate analysis. No clinically relevant episodes of hypotension were observed in this study [32]. Finally, among 628 patients with a total of 1,100 large volume paracenteses performed, mean pre-procedural INR was 1.7 (0.9-8.7) and platelets were 50,400/μL (19,000-341,000/μL), without any procedure-related complications [45]. These data demonstrate the safety of paracentesis in the cirrhotic population, as supported by clinical guidelines [25].

Volume Expanders

A therapeutic, or large-volume paracentesis, is indicated in a hemodynamically stable patient with tense ascites and respiratory distress or with significant physical discomfort. Paracentesis-induced circulatory dysfunction (PICD) is a potentially harmful complication of large volume paracentesis, associated with faster re-accumulation of ascites, renal impairment, and shorter survival; therefore, plasma expanders are recommended for large-volume paracentesis (>4 L) [46]. In a randomized trial of 72 patients, albumin was more effective at preventing paracentesis-induced circulatory dysfunction as compared to saline when evacuating 6 L or more of ascitic fluid [47].

Disposition

Providers should maintain a low threshold for performing a paracentesis in any cirrhotic patient who presents to the ED with ascites and abdominal pain or tenderness, altered mental status, or fever [18]. Furthermore, all patients with ascites who are admitted to the hospital should have a paracentesis performed and treated with antibiotics if there are >250/mL PMN. Given that up to half of cirrhotic patients present to the ED with bacterial infections, or develop them after admission [14], a high clinical suspicion is warranted. Observation or admission should be considered in patients with any clinical signs of infection, even with normal ascetic fluid.

## Conclusions

SBP has a high mortality rate, and early diagnosis and antimicrobial therapy are essential for improving patient outcomes. Any cirrhotic patient with ascites with clinical suspicion for SBP, abdominal pain or tenderness, altered mental status or fever should have a diagnostic paracentesis performed prior to admission. Timely antimicrobial therapy should include a third-generation cephalosporin for community-acquired infection, and nosocomial infections should be treated empirically based on local susceptibility testing of bacteria. Albumin should also be administered when indicated based on laboratory studies. The current recommendation for prophylactic antibiotics for GI bleed is ceftriaxone. Although the majority of patients with cirrhosis and liver dysfunction will have prolonged prothrombin time, paracentesis is not contraindicated in patients with coagulopathy, but consider giving platelets prior to paracentesis if <40,000-50,000/μL.

## References

[1] Pant C, Olyaee M, Gilroy R (2015). Emergency department visits related to cirrhosis: a retrospective study of the nationwide emergency department sample 2006 to 2011. Medicine.

[2] CDC CDC (2017). Hepatitis C FAQs for health professionals. Department of Health and Human Services.

[3] Caly WR, Strauss E (1993). A prospective study of bacterial infections in patients with cirrhosis. J Hepatol.

[4] Tsochatzis EA, Bosch J, Burroughs AK (2014). Liver cirrhosis. Lancet.

[5] D'Amico G, Garcia-Tsao G, Pagliaro L (2006). Natural history and prognostic indicators of survival in cirrhosis: a systematic review of 118 studies. J Hepatol.

[6] D'Amico G, Pasta L, Morabito A (2014). Competing risks and prognostic stages of cirrhosis: a 25-year inception cohort study of 494 patients. Aliment Pharmacol Ther.

[7] Leber B, Spindelboeck W, Stadlbauer V (2012). Infectious complications of acute and chronic liver disease. Semin Respir Crit Care Med.

[8] Tsiaoussis GI, Assimakopoulos SF, Tsamandas AC (2015). Intestinal barrier dysfunction in cirrhosis: Current concepts in pathophysiology and clinical implications. World J Hepatol.

[9] Arvaniti V, D'Amico G, Fede G (2010). Infections in patients with cirrhosis increase mortality four-fold and should be used in determining prognosis. Gastroenterology.

[10] Garcia-Tsao G (1992). Spontaneous bacterial peritonitis. Gastroenterol Clin North Am.

[11] Goulis J, Armonis A, Patch D (1998). Bacterial infection is independently associated with failure to control bleeding in cirrhotic patients with gastrointestinal hemorrhage. Hepatology.

[12] Bernard B, Cadranel JF, Valla D (1995). Prognostic significance of bacterial infection in bleeding cirrhotic patients: a prospective study. Gastroenterology.

[13] Corey KE, Friedman LS (2015). Abdominal swelling and ascites. Harrison's Principles of Internal Medicine.

[14] De Mattos AA, Costabeber AM, Lionco LC (2014). Multi-resistant bacteria in spontaneous bacterial peritonitis: a new step in management?. World J Gastroenterol.

[15] De Mattos AA, Coral G, Menti E (2003). Bacterial infection in cirrhotic patient. Arq Gastroenterol.

[16] Chinnock B, Afarian H, Minnigan H (2008). Physician clinical impression does not rule out spontaneous bacterial peritonitis in patients undergoing emergency department paracentesis. Ann Emerg Med.

[17] Shi KQ, Fan YC, Ying L (2012). Risk stratification of spontaneous bacterial peritonitis in cirrhosis with ascites based on classification and regression tree analysis. Mol Biol Rep.

[18] Wehmeyer MH, Krohm S, Kastein F (2014). Prediction of spontaneous bacterial peritonitis in cirrhotic ascites by a simple scoring system. Scand J of Gastroenterology.

[19] Bacon BR (2015). Cirrhosis and its complications. Harrison's Principles of Internal Medicine.

[20] Baraldi O, Valentini C, Donati G (2015). Hepatorenal syndrome: update on diagnosis and treatment. World J Nephrology.

[21] Friedman G, Berlot G, Kahn RJ (1995). Combined measurements of blood lactate concentrations and gastric intramucosal pH in patients with severe sepsis. Crit Care Med.

[22] Gaetano JN, Micic D, Aronsohn A (2016). The benefit of paracentesis on hospitalized adults with cirrhosis and ascites. J Gastroenterol Hepatol.

[23] Kim JJ, Tsukamoto MM, Mathur AK (2014). Delayed paracentesis is associated with increased in-hospital mortality in patients with spontaneous bacterial peritonitis. Am J Gastroenterol.

[24] Albillos A, Cuervas-Mons V, Millan I (1990). Ascitic fluid polymorphonuclear cell count and serum to ascites albumin gradient in the diagnosis of bacterial peritonitis. Gastroenterology.

[25] Runyon BA, AASLD Practice Guidelines Committee (2017). Management of adult patients with ascites due to cirrhosis: an update. Hepatology.

[26] Sakai H, Sheer TA, Mendler MH (2005). Choosing the location for non-image guided abdominal paracentesis. Liver Int.

[27] Mercaldi CJ, Lanes SF (2013). Ultrasound guidance decreases complications and improves the cost of care among patients undergoing thoracentesis and paracentesis. Chest.

[28] Nazeer SR, Dewbre H, Miller AH (2005). Ultrasound-assisted paracentesis performed by emergency physicians vs the traditional technique: a prospective, randomized study. Am J Emerg Med.

[29] Edell SL, Gefter WB (1979). Ultrasonic differentiation of types of ascitic fluid. AJR Am J Roentgenol.

[30] Lin CH, Shih FY, Ma MHM (2005). Should bleeding tendency deter abdominal paracentesis?. Dig Liver Dis.

[31] Hibbert R, Atwell T, Lekah A (2013). Safety of ultrasound-guided thoracentesis in patients with abnormal preprocedural coagulation parameters. Chest.

[32] De Gottardi A, Thevenot T, Spahr L (2009). Risk of complications after abdominal paracentesis in cirrhotic patients: a prospective study. Clin Gastroenterol Hepatol.

[33] Mallory A, Schaefer JW (1978). Complications of diagnostic paracentesis in patients with liver disease. JAMA.

[34] Karvellas CJ, Abraldes J, Arabi YM (2015). Appropriate and timely antimicrobial therapy in cirrhotic patients with spontaneous bacterial peritonitis-associated septic shock: a retrospective cohort study. Aliment Pharmacol Ther.

[35] Friedrich K, Nussle S, Rehlen T (2016). Microbiology and resistence in first episodes of spontaneous bacterial peritonitis: implications for management and prognosis. J Gastroenterol Hepatol.

[36] Falcone M, Russo A, Pacini G (2015). Spontaneous bacterial peritonitis due to methicillin-resistant S. aureus in a patient with cirrhosis: the potential role for daptomycin and review of the literature. Infect Dis Rep.

[37] Sort P, Navasa M, Arroyo V (1999). Effect of intravenous albumin on renal impairment and mortality in patients with cirrhosis and spontaneous bacterial peritonitis. N Engl J Med.

[38] Bleichner G, Boulanger R, Squara P (1986). Frequency of infections in cirrhotic patients presenting with acute gastrointestinal haemorrhage. Br J Surg.

[39] Pauwels A, Mostefa-Kara N, Debenes B (1996). Systemic antibiotic prophylaxis after gastrointestinal hemorrhage in cirrhotic patients with a high risk of infection. Hepatology.

[40] Fernandez J, Ruiz del Arbol L, Gomez C (2006). Norfloxacin vs ceftriaxone in the prophylaxis of infections in patients with advanced cirrhosis and hemorrhage. Gastroenterology.

[41] Dever JB, Sheikh MY (2015). Review article: spontaneous bacterial peritonitis--bacteriology, diagnosis, treatment, risk factors and prevention. Aliment Pharmacol Ther.

[42] Moore KP, Aithal GP (2006). Guidelines on the management of ascites in cirrhosis. Gut.

[43] Kurup AN, Lekah A, Reardon ST (2015). Bleeding rate for ultrasound-guided paracentesis in thrombocytopenic patients. J Ultrasound Med.

[44] Grabau CM, Crago S, Hoff LK (2004). Performance standards for therapeutic abdominal paracentesis. Hepatology.

[45] Gines A, Fernandez-Esparrach G, Monescillo A (1996). Randomized trial comparing albumin, dextran 70, and polygeline in cirrhotic patients with ascites treated by paracentesis. Gastroenterology.

[46] Sola-Vera J, Minana J, Ricart E (2003). Randomized trial comparing albumin and saline in the prevention of paracentesis-induced circulatory dysfunction in cirrhotic patients with ascites. Hepatology.

[47] Jamtgaard L, Manning SL, Cohn B (2016). Does albumin infusion reduce renal impairment and mortality in patients with spontaneous bacterial peritonitis?. Ann Emerg Med.

